# Assessment of bone marrow fat fractions in the mandibular condyle head using the iterative decomposition of water and fat with echo asymmetry and least-squares estimation (IDEAL-IQ) method

**DOI:** 10.1371/journal.pone.0246596

**Published:** 2021-02-26

**Authors:** Kug Jin Jeon, Chena Lee, Yoon Joo Choi, Sang-Sun Han

**Affiliations:** Department of Oral and Maxillofacial Radiology, Yonsei University College of Dentistry, Seoul, Republic of Korea; Thamar University, Faculty of Dentistry, YEMEN

## Abstract

The prevalence of temporomandibular joint disorder (TMD) is gradually increasing, and magnetic resonance imaging (MRI) is becoming increasingly common as a modality used to diagnose TMD. Edema and osteonecrosis in the bone marrow of the mandibular condyle have been considered to be precursors of osteoarthritis, but these changes are not evaluated accurately and quantitatively on routine MRI. The iterative decomposition of water and fat with echo asymmetry and least-squares estimation (IDEAL-IQ) method, as a cutting-edge MRI technique, can separate fat and water using three asymmetric echo times and the three-point Dixon method. The purpose of this study was to analyze the quantitative fat fraction (FF) in the mandibular condyle head using the IDEAL-IQ method. Seventy-nine people who underwent MRI using IDEAL-IQ were investigated and divided into 1) the control group, without TMD symptoms, and 2) the TMD group, with unilateral temporomandibular joint (TMJ) pain. In both groups, the FF of the condyle head in the TMJ was analyzed by two oral and maxillofacial radiologists. In the TMD group, 29 people underwent cone-beam computed tomography (CBCT) and the presence or absence of bony changes in the condylar head was evaluated. The FF measurements of the condyle head using IDEAL-IQ showed excellent inter-observer and intra-observer agreement. The average FF of the TMD group was significantly lower than that of the control group (*p* < 0.05). In the TMD group, the average FF values of joints with pain and joints with bony changes were significantly lower than those of joints without pain or bony changes, respectively (*p* < 0.05). The FF using IDEAL-IQ in the TMJ can be helpful for the quantitative diagnosis of TMD.

## Introduction

The prevalence of temporomandibular joint disorder (TMD) is gradually increasing, and cone-beam computed tomography (CBCT), computed tomography (CT) and magnetic resonance imaging (MRI) are becoming increasingly common as modalities used to diagnose TMD.

CT is an excellent imaging modality for evaluating bony changes of the temporomandibular joint (TMJ) and can be expressed by quantifying the density of tissue using Hounsfield units (HU), which are relatively set based on the value of -1000 for air and 0 for water. MRI is excellent for evaluating soft tissue. It shows the soft tissue, including the disc of the TMJ and its attachment, as well as joint effusion and the bone marrow signal of the condylar head [1]. MRI does not use radiation, and new advanced techniques for diagnosis are being developed, including higher magnetic field strength, improved coil design, and other technical improvements [2], resulting in increasing demand for MRI. Various MRI techniques have been tried in recent years, and for the TMJ, studies have been conducted using dynamic MRI [3], real-time MRI [4, 5], zero-TE technique [6], and T2 mapping [7]. The bone marrow consists of water, fat, and protein, and its proportion is thought to be related to the remodeling capacity of the bone [8]. In previous studies, edema and osteonecrosis in the bone marrow of the mandibular condyle were considered to be a precursor to osteoarthritis, but these changes were not evaluated accurately and quantitatively on routine MRI [5, 9–11]. One of the latest MRI techniques is the iterative decomposition of water and fat with echo asymmetry and least-squares estimation (IDEAL-IQ) method, which can separate fat and water using three asymmetric echo times and the three-point Dixon method [12]. IDEAL-IQ has been studied for the liver [13–16], pancreas [17], femur [8], and salivary glands [18], as well as for the diagnosis of osteoporosis [8] and metabolic diseases such as diabetes mellitus [19, 20] through changes in the fat fraction (FF). Yoo et al. [21] reported that malignant lesions replaced normal bone marrow fat with cancer cells, resulting in a reduced percentage of fat. However, the utility of IDEAL-IQ for the TMJ has not yet been assessed.

This study quantitatively measured the FF in the condyle head of the TMJ using the IDEAL-IQ method and statistically compared the control and TMD groups. Further, the FF was compared within the TMD group according to the presence of pain and bony changes to assess its utility in the accurate diagnosis of TMD.

## Materials and methods

### Subject classification

Seventy-nine patients who underwent MRI examinations including a TMJ IDEAL-IQ sequence from April 2019 to June 2020 at Yonsei University Dental Hospital were retrospectively selected.

We classified patients without any TMD symptoms into the control group and those with TMD symptoms into the TMD group. Symptoms were defined as pain, sound (click, crepitus, or popping), and limitation of mouth opening (assisted opening < 40 mm) according to the Diagnostic Criteria for Temporomandibular Disorders (DC/TMD) [22]. The TMD group included only patients with unilateral TMJ pain.

The control group consisted of 34 people (68 joints) (15 male and 19 female; age range, 35–79 years; mean age, 59.44 years). Forty-five people (90 joints) with TMD symptoms were classified into the TMD group (13 male and 32 female; age range, 32–76 years; mean age, 48.40 years).

### Quantitative analysis of MRI

**Fig 1:**
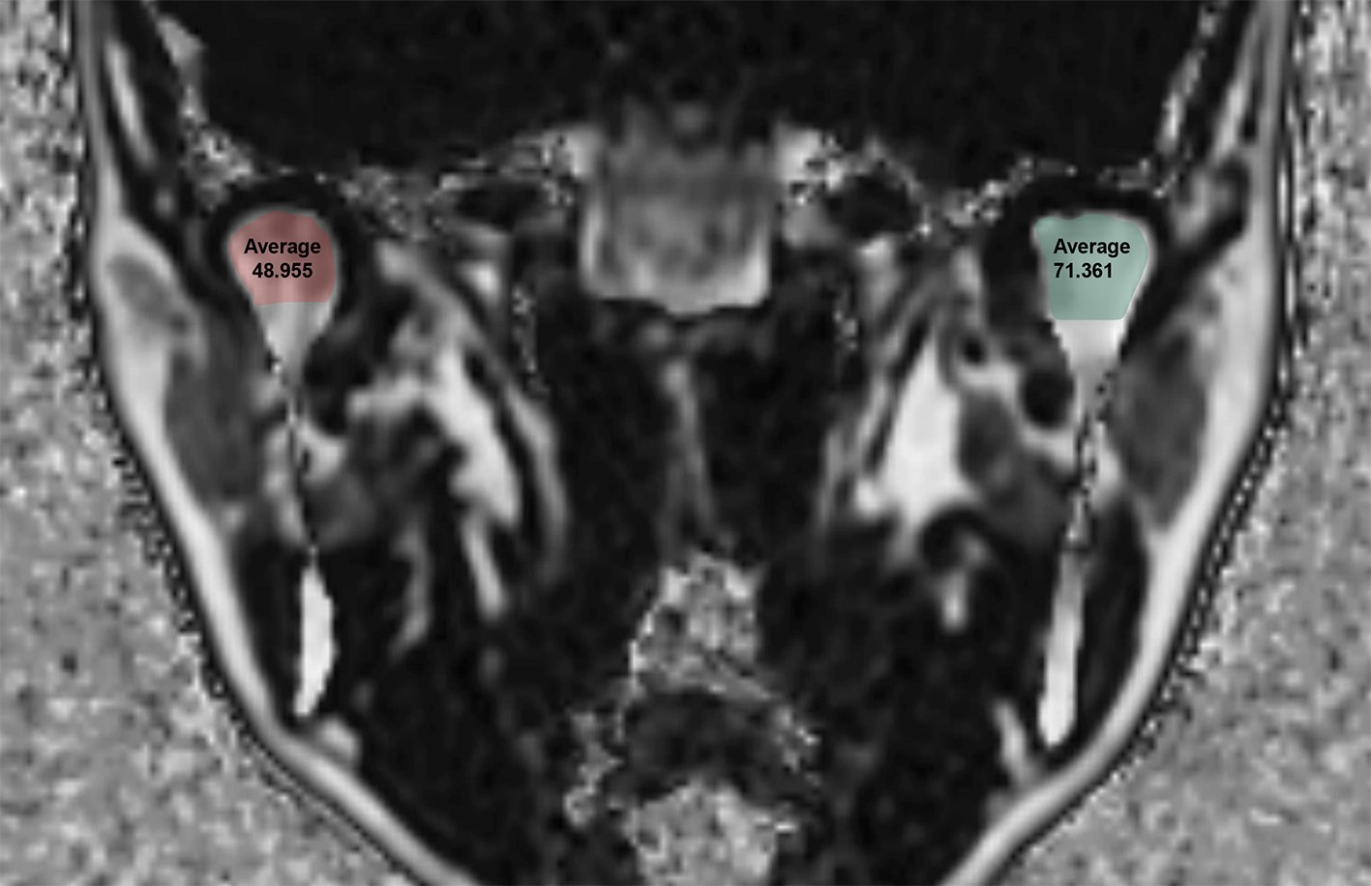
Fat Fraction (FF) using the IDEAL-IQ sequence in a 43-year-old female patient with temporomandibular joint disorder.

MRI of the TMJ was acquired using a 3.0 T scanner (Pioneer; GE Healthcare, Waukesha, WI, USA) with 16-channel flex large coil. Images of the 3D coronal IDEAL-IQ sequence were acquired using the following parameters: echo time, 9.9 ms; repetition time, 6.5 ms; bandwidth, 111.11 kHz; NEX, 1.0; field of view, 240 × 240 mm; slice thickness, 2.0 mm; scan time, 1 min. The FF of the TMJ as determined using IDEAL-IQ was analyzed in the control group and TMD group. Regions of interest were drawn along the inner surface of the cortical layer to the condylar neck on the coronal image where the condylar head was most visible (Fig 1). Oral and maxillofacial radiologists with 20 years of experience analyzed of FF twice at 1 month intervals. The average values of two measurements were used for the statistical analysis. To assess reliability, 20 MRI images (40 joints) were randomly selected and oral and maxillofacial radiologists with 12 years of experience analyzed of FF twice at 1 month intervals.

### Bony change evaluation of the TMD group on CBCT images

In the TMD group, 29 people underwent both MRI and CBCT examinations within 2 months (10 male and 19 female; age range, 36–74 years; mean age, 47.69 years). Two oral and maxillofacial radiologist assessed bony changes in the mandibular condyle. Bony change was defined as the presence of erosion (an interruption or absence of the cortical lining) or an osteophyte (marginal hypertrophic bone formation) according to the criteria proposed by Shigeno et al. [7]. The presence or absence of bony changes was defined by the consensus of two radiologists.

### Statistical analysis

The intra- and inter-observer agreement of the two radiologists was assessed using kappa analysis. The FF between the control group and the TMD group was compared using the unpaired t-test. In the TMD group with unilateral joint pain, the FF between the painful joint and the painless joint were compared using the paired t-test, and the FF between joints with and without bony changes were compared using the unpaired t-test. Statistical analyses were performed using GraphPad Prism version 8.0 (GraphPad Software, La Jolla, CA, USA).

### Ethics approval

The study was approved by the Institutional Review Board of Yonsei University Dental Hospital (IRB no. 2-2020-0014) and was performed in accordance with all relevant guidelines and regulations. The requirement for patient consent was waived because of the retrospective nature of the study.

## Results

The kappa coefficients for intra-observer agreement were 0.898 (95% confidence interval [CI], 0.807–0.946) and 0.993 (95% CI, 0.987–0.996), respectively. The inter-observer agreement of the two observers was 0.960 (95% CI, 0.924–0.979) (Table 1).

**Table 1:** Intra- and inter-observer agreement for two radiologists: Kappa index (95% CI).

	Radiologist 1	Radiologist 2
**Intra-observer agreement**	0.898 (0.807–0.946)	0.993(0.987–0.996)
**Inter-observer agreement**	0.960 (0.924–0.979)	

CI, confidence interval.

**Fig 2:**
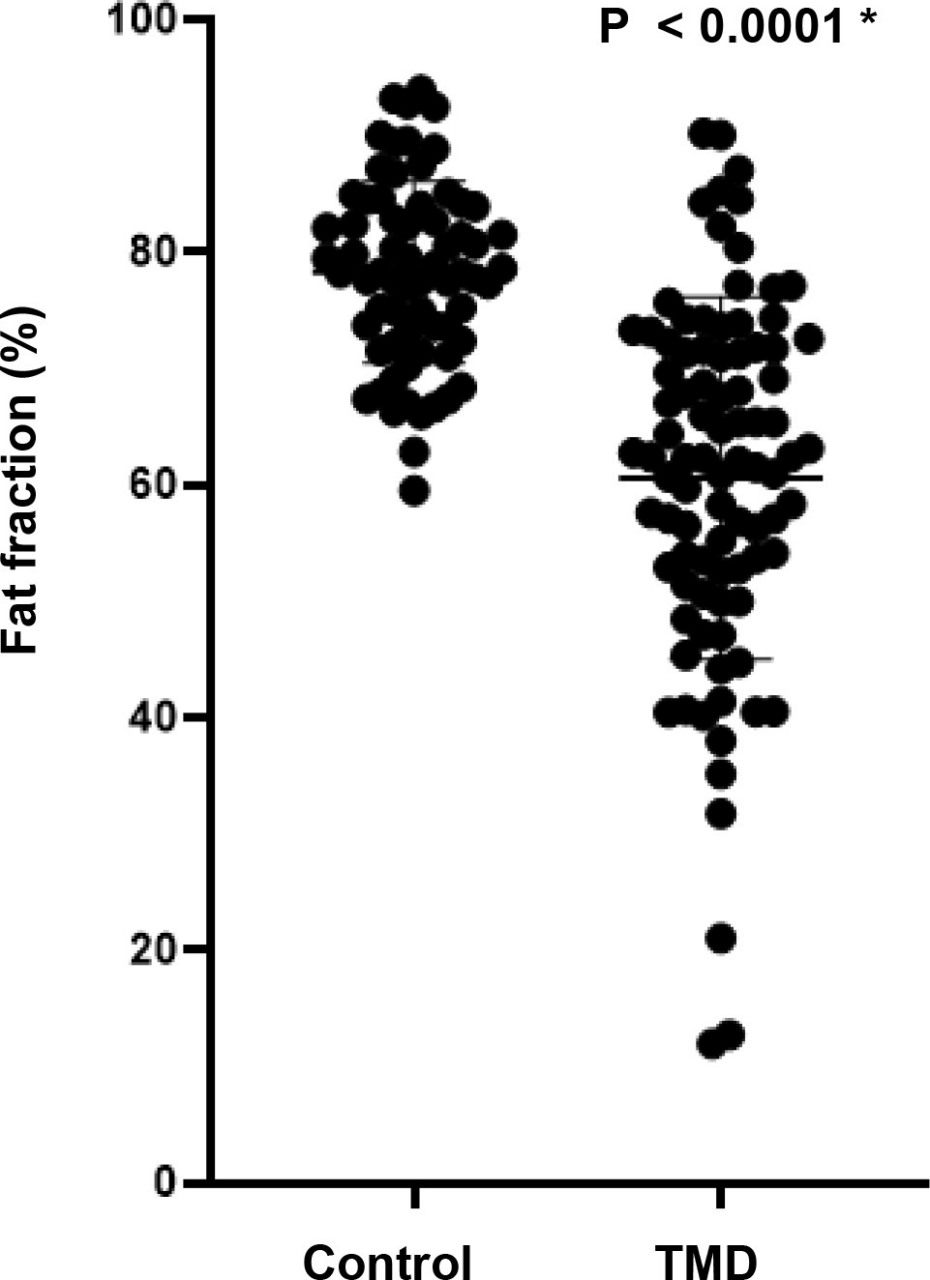
Fat fraction (%) between the control group and Temporomandibular Joint Disorder (TMD) group.

The average FF value of the TMD group was 60.56%, which was statistically significantly lower than that of the control group (*p*-value<0.0001) (Table 2 and Fig 2). Detailed FF values for the control and TMD groups are shown in S1 and S2 Tables.

**Table 2:** Fat fraction (%) between the control group and Temporomandibular Joint Disorder (TMD) group.

Control group	TMD group	Difference (95% CI)	*p*-value
(n = 68 joints)	(n = 90 joints)		
**78.30**	60.56	17.73 ± 2.07 (13.66–21.81)	< 0.0001 *

CI, confidence interval; unpaired t-test, * *p*<0.05.

**Fig 3:**
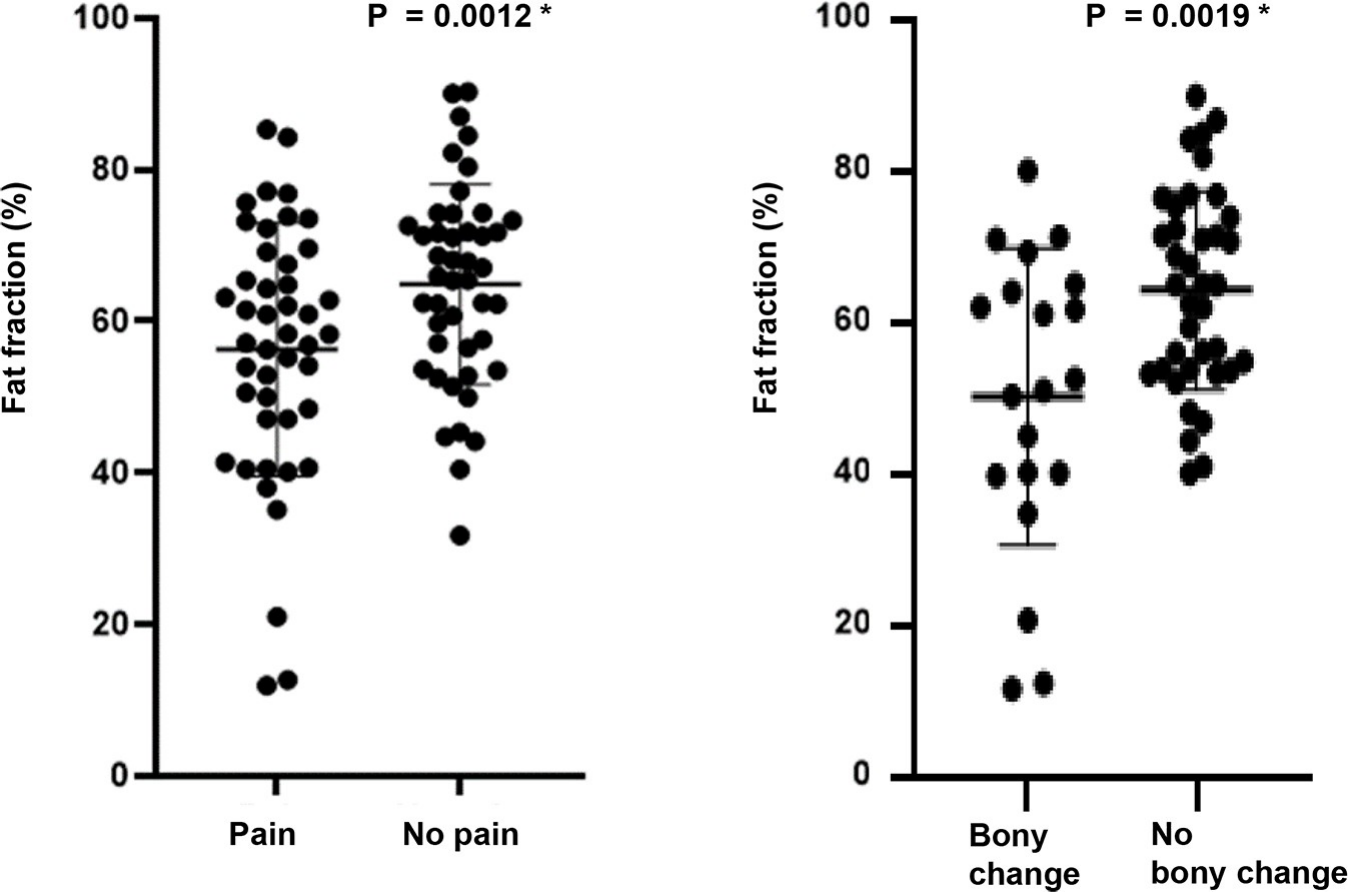
Fat fraction (%) according to the presence of pain and bony changes within the TMD group.

Within the TMD group, the average FF value of painful joints was 55.98%, which was statistically significantly lower than that of painless joints (*p*-value = 0.0012), and the average FF value of joints with bony changes was 50.54%, which was statistically significantly lower than that of joints without bony changes (*p*-value = 0.0019) (Table 3 and Fig 3).

**Table 3:** Fat fraction (%) according to the presence of pain and bony changes in the TMD group.

	Yes	No	Difference (95% CI)	*p*-value
**Pain**	55.98	64.49	8.58 ± 16.67 (3.58–13.59)	0.0012 *
	(n = 45 joints)	(n = 45 joints)		
**Bony changes**	50.54	64.62	14.08 ± 4.32 (5.43–22.73)	0.0019 *
	(n = 20 joints)	(n = 38 joints)		

CI, confidence interval; pain, paired t-test, *p<0.05; bony changes, unpaired t-test, *p<0.05.

## Discussion

TMD is a chronic disease, in which patients can undergo cycles of symptom improvement and exacerbation. It would be very helpful if imaging provided indicators that could predict a patient’s future prognosis beyond just showing the current status of bony changes or disc condition.

Edema and osteonecrosis may occur in the marrow of the mandibular condyle. Osteoarthritis may be secondary to osteonecrosis in the TMJ, and edema may be a precursor to osteonecrosis or a sign of worsening of symptoms [9]. It is known that osteonecrosis manifests as low signal intensity on T1-weighted MRI, while edema is displayed as high signal intensity on T2-weighted images [23, 24], but these signal changes are subjectively judged by visual inspection. Thus, an objective and quantifiable evaluation method is needed.

The red (hematopoietic) marrow is composed of 40% fat, 40% water, and 20% protein, and the yellow (fatty) marrow in adults is composed of approximately 80% fat, 15% water, and 5% protein [25]. The proportion of both types of marrow appears to be related to the remodeling capacity of bone. Therefore, it is thought that changes of the FF in the condylar bone marrow may help predict the progression of TMD.

Several MRI techniques can be used to quantify FF. MR spectroscopy can accurately measure fat-water content [26, 27], but due to its long scan time, small image range, and post-processing, it has limitations in clinical applications [13, 28]. The latest IDEAL-IQ technique can obtain FF images without post-processing, and can automatically calculate the FF with shorter scan and measuring time [8, 29]. Meisamy et al. [14] reported the accuracy of IDEAL-IQ in the liver and Aoki et al. [8] reported its reproducibility in pelvic imaging through 10 repeated evaluations. Aoki et al. [8] also reported that the FF significantly increased with age and that the FF of postmenopausal women was significantly higher than that of premenopausal women in the lumbar vertebral bodies, ilium, and intertrochanteric region of the femur. Hu et al. [19] reported that IDEAL-IQ findings were highly associated with increasing vertebral fat deposition and that IDEAL-IQ could be used to quantitatively evaluate changes in fat deposition in alloxan-induced diabetic rabbits. Chen et al. [20] reported that pancreatic fat infiltration was associated with diabetes and that IDEAL-IQ could be used to accurately and reproducibly quantify pancreatic fat content in 7 pigs with diabetes and 6 control pigs. In the intervertebral disc of the vertebra, the signal intensity of the focal lesion was evaluated by visual assessment in T1-weighted images to distinguish normal hematopoietic marrow and infiltrative pathology [30, 31]. A report suggested that malignant lesions of the spine have lower FF than benign lesions, and that the FF could therefore be useful for distinguishing between benign and malignant causes of focal bone marrow abnormalities [21]. It is also thought that in TMD, the TMJ has FF changes, but to the best of our knowledge, no studies have yet investigated the application of IDEAL-IQ in TMJ.

In this study, the FF measurements of the condyle head using IDEAL-IQ showed excellent intra- and inter-observer agreement. The FF was statistically significantly lower in the TMD group than in the control group. In patients with TMD, the FF is thought to decrease due to the loss of normal bone marrow fat, increased serum protein exudate, and edema fluid [9]. According to the three-way histologic classification of core biopsies of the mandibular condyle by Larheim et al. [9], normal samples show a normal marrow architecture in which the hematopoietic marrow is maintained, while samples with edema show preservation of the hematopoietic bone marrow and production of serum protein exudate and edema fluid within the marrow interstitium. Finally, samples with osteonecrosis display loss of the hematopoietic marrow, the normal marrow stroma, and marrow fat, as well as gradual reticulin fibrosis.

Fat content in the TMD group was statistically significantly lower in the joints with pain and bony changes than those without pain or bony changes. TMJ pain can have a variety of sources, including inflammatory changes in the posterior disc attachment [32], the presence of inflammatory mediators in the synovial fluid [33], excessive joint effusion [34, 35], and bone marrow edema [9, 35–41]. It has been reported that the risk and degree of pain in TMJs with an abnormal bone marrow signal were significantly higher than those in TMJs with a normal bone marrow signal on MRI [37, 42]. If there are bony changes, the bone marrow may be lost in addition to the cortical bone, which may be accompanied by edema and osteonecrosis; therefore, the fat content decreases. In light of the observed difference in the FF value between the TMD group and the control group, it is noteworthy that a recent study showed the same tendency in the sacroiliac joint. The mean FF of normal sacroiliac joints was 52.0 ± 10.4% and 50.5 ± 10.1% on the left and right sides, respectively, whereas active inflammatory fat deposition in the patients with spondyloarthritis led to a much lower FF: 15.8 ± 5.9% and 13.5 ± 6.7% on the left and right sides, respectively. However, post-inflammatory FF indicating chronicity was higher than normal [43]. In TMJ, further studies are needed according to the stage of the disease.

The FF of the bone marrow can have different values depending on age, sex, and body mass index (BMI). Previous studies have shown that FF tends to increase gradually with age in the parotid glands, submandibular glands, and sacroiliac joints [18, 44]. At birth, the mandible only contains red marrow, but with age, conversion of red marrow to yellow marrow begins anteriorly, and by age 30, only yellow marrow is found in the normal mandible [45]. Therefore, this study targeted people over the age of 30. The TMD group and the control group were designed to include patients in similar age groups to minimize the effect of age. Previous studies showed no statistically significant differences between male and female [18, 43]. The reported relationship between FF and BMI has varied across studies, with the salivary gland being related, but the sacroiliac joint not [18, 43]. Further studies on the FF of the mandibular condyle according to age, sex, and BMI are needed. In addition, since the mandibular condyle is a site that is loaded by occlusion, further studies on the relationship with occlusal force are also required.

The main limitation of this study is the number of samples, and since bony changes only included erosion and osteophytes, further studies on minor bony changes such as sclerosis, flattening, and subchondral cyst are needed. Further research is also needed on the relationship between intensity of pain and FF values. Finally, we did not consider systemic conditions such as drug treatment, radiation treatment, and metabolic disorders.

In conclusion, the FF, assessed quantitatively using IDEAL-IQ in the TMJ, was lower in patients with TMD than in the control group, and there were significant differences depending on the presence or absence of pain and bony changes in the TMD group. IDEAL-IQ could be used to diagnose TMD on MRI based on a quantitative assessment of the FF of the condylar bone marrow.

## Supporting information

S1 Table Control group. (DOCX)

S2 Table TMD group. (DOCX)
