# Supplementary material for: Assessment of bone marrow fat fractions in the mandibular condyle head using the iterative decomposition of water and fat with echo asymmetry and least-squares estimation (IDEAL-IQ) method
Source: PLoS One. 2021 Feb 26;16(2):e0246596. doi: 10.1371/journal.pone.0246596 (PMC7909693; doi:10.1371/journal.pone.0246596)
Supplement: S1 Table — (DOCX) [file pone.0246596.s001.docx]

S1 Table. Control group

| Patient  No. | Age  (years) | Sex | Fat fraction (%) | |
| --- | --- | --- | --- | --- |
|  |  |  | Right TMJ | Left TMJ |
| 1 | 35 | M | 83.9815 | 77.446 |
| 2 | 40 | F | 77.77 | 75.153 |
| 3 | 45 | F | 62.8225 | 66.3185 |
| 4 | 48 | M | 76.982 | 82.6825 |
| 5 | 48 | F | 79.801 | 77.5355 |
| 6 | 48 | F | 67.3735 | 59.5325 |
| 7 | 49 | M | 72.0305 | 66.67 |
| 8 | 50 | F | 74.946 | 78.22 |
| 9 | 52 | M | 78.25 | 82.824 |
| 10 | 53 | M | 66.9505 | 66.057 |
| 11 | 53 | F | 80.537 | 84.595 |
| 12 | 54 | F | 82.3545 | 84.53 |
| 13 | 57 | M | 71.357 | 70.1785 |
| 14 | 57 | M | 71.516 | 68.4015 |
| 15 | 58 | M | 78.573 | 80.7125 |
| 16 | 58 | F | 77.608 | 79.522 |
| 17 | 60 | F | 68.9995 | 67.882 |
| 18 | 60 | M | 84.9175 | 93.2025 |
| 19 | 61 | F | 73.7245 | 78.8385 |
| 20 | 61 | F | 77.3015 | 82.3565 |
| 21 | 62 | F | 71.075 | 75.2465 |
| 22 | 62 | F | 86.777 | 87.148 |
| 23 | 64 | F | 83.9115 | 85.0775 |
| 24 | 65 | M | 72.3645 | 73.5935 |
| 25 | 66 | M | 92.7585 | 93.9185 |
| 26 | 67 | M | 90.0345 | 80.2675 |
| 27 | 68 | F | 77.5365 | 78.2345 |
| 28 | 69 | F | 88.8825 | 82.0795 |
| 29 | 71 | F | 89.576 | 89.37 |
| 30 | 73 | F | 81.488 | 84.242 |
| 31 | 73 | M | 75.006 | 81.382 |
| 32 | 76 | M | 92.494 | 87.521 |
| 33 | 79 | F | 73.275 | 79.4785 |
| 34 | 79 | M | 67.3645 | 73.618 |

Abbreviations: M, male; F, female
