# Supplementary material for: Assessment of bone marrow fat fractions in the mandibular condyle head using the iterative decomposition of water and fat with echo asymmetry and least-squares estimation (IDEAL-IQ) method
Source: PLoS One. 2021 Feb 26;16(2):e0246596. doi: 10.1371/journal.pone.0246596 (PMC7909693; doi:10.1371/journal.pone.0246596)
Supplement: S2 Table — (DOCX) [file pone.0246596.s002.docx]

S2 Table. TMD group

| Patient No. | Age  (years) | Sex | Right TMJ | | | Left TMJ | | |
| --- | --- | --- | --- | --- | --- | --- | --- | --- |
|  |  |  | Fat fraction (%) | Bony changes | Fat fraction (%) | | Bony changes | |
| 1 | 32 | M | 73.3065 |  | **73.182** | |  | |
| 2 | 33 | M | **58.2575** |  | 31.729 | |  | |
| 3 | 36 | M | 62.249 |  | **60.872** | |  | |
| 4 | 36 | M | **69.1545** | X | 71.7615 | | X | |
| 5 | 36 | F | **55.1715** | X | 56.4765 | | X | |
| 6 | 37 | F | **58.3235** |  | 57.0605 | |  | |
| 7 | 38 | F | 53.66 | X | **53.9405** | | X | |
| 8 | 38 | M | **85.2915** | X | 90.231 | | X | |
| 9 | 40 | F | 80.388 | O | **21.0445** | | O | |
| 10 | 40 | F | **40.1365** | O | 52.4795 | | X | |
| 11 | 41 | F | **40.5225** | O | 62.412 | | O | |
| 12 | 41 | F | 59.729 | X | **62.7655** | | X | |
| 13 | 42 | F | 74.191 | X | **62.0175** | | O | |
| 14 | 42 | F | 71.628 | O | **50.593** | | O | |
| 15 | 43 | F | 40.4605 | X | **41.3785** | | X | |
| 16 | 43 | F | **49.9875** |  | 68.6235 | |  | |
| 17 | 43 | F | **67.4875** |  | 49.936 | |  | |
| 18 | 44 | M | 77.1675 | X | **12.7025** | | O | |
| 19 | 44 | F | 65.3975 | X | **76.8095** | | X | |
| 20 | 45 | F | 57.5695 |  | **47.087** | |  | |
| 21 | 46 | F | **11.952** | O | 62.2785 | | X | |
| 22 | 46 | F | 45.358 | O | **40.4835** | | O | |
| 23 | 46 | M | **75.6305** | X | 82.198 | | X | |
| 24 | 46 | F | **56.844** | X | 71.3105 | | X | |
| 25 | 47 | F | 65.966 | X | **54.146** | | X | |
| 26 | 48 | F | **57.1265** | X | 65.371 | | X | |
| 27 | 48 | F | 53.515 | X | **48.4585** | | X | |
| 28 | 48 | M | **60.935** |  | 67.0475 | |  | |
| 29 | 49 | F | 51.405 | O | **35.1385** | | O | |
| 30 | 49 | F | **40.66** |  | 52.8065 | |  | |
| 31 | 49 | F | **38.0105** |  | 44.1635 | |  | |
| 32 | 49 | F | 44.742 | X | **47.123** | | X | |
| 33 | 49 | F | **69.5985** | O | 72.6045 | | X | |
| 34 | 52 | M | 90.052 |  | **84.25** | |  |  |
| 35 | 53 | M | **77.1095** | X | 84.5065 | | X |  |
| 36 | 60 | M | **56.291** | X | 67.8365 | | X |  |
| 37 | 61 | F | **65.364** | O | 71.81 | | X |  |
| 38 | 61 | F | 62.413 |  | **73.865** | |  |  |
| 39 | 63 | F | **61.492** | O | 71.2575 | | O |  |
| 40 | 63 | M | **73.5305** |  | 74.2325 | |  |  |
| 41 | 64 | F | **72.2675** |  | 68.078 | |  |  |
| 42 | 64 | F | **64.8015** |  | 74.2975 | |  |  |
| 43 | 73 | M | **64.3365** | O | 87.0215 | | X |  |
| 44 | 74 | F | **52.9165** | O | 71.0275 | | X |  |
| 45 | 76 | F | **63.116** |  | 60.7115 | |  |  |

Abbreviations: M, male; F, female

Pain side is bold.
